# Shenlian extract attenuates myocardial ischaemia-reperfusion injury via inhibiting M1 macrophage polarization by silencing *miR-155*

**DOI:** 10.1080/13880209.2022.2117828

**Published:** 2022-10-14

**Authors:** Min Song, Xihe Cui, Jing Zhang, Yujie Li, Jingjing Li, Yuanlong Zang, Qi Li, Qing Yang, Ying Chen, Weiyan Cai, Xiaogang Weng, Yajie Wang, Xiaoxin Zhu

**Affiliations:** Institute of Chinese Materia Medica, China Academy of Chinese Medical Science, Beijing, China

**Keywords:** MI/RI, apoptosis, *Salvia miltiorrhiza*, *Andrographis paniculata*

## Abstract

**Context:**

Shenlian extract (SL) is a combination of *Salvia miltiorrhiza* Bge. (Labiatae) and *Andrographis paniculata* (Burm. F.) Wall. Ex Nees (Acanthaceae) extracts, which promote blood circulation and clear endogenous heat toxins. Myocardial ischaemia-reperfusion injury (MI/RI) is aggravated myocardial tissue damage induced by reperfusion therapy after myocardial infarction.

**Objectives:**

This study explores the effect of SL on MI/RI and the underlying mechanism.

**Materials and methods:**

Primary peritoneal macrophages (pMACs) were treated with LPS and SL (5, 10 or 20 μg/mL) for 24 h. The myocardial ischaemia-reperfusion (MI/R) model was established after administration of different doses of SL (90, 180 or 360 mg/kg). Myocardial tissue injury was assessed by methylthiazolyl tetrazolium (TTC) staining and levels of creatine kinase (CK), lactate dehydrogenase (LDH) and superoxide dismutase (SOD) in mice. The double immunofluorescence staining of iNOS/F4/80 and CD86/F4/80 was used to detect macrophage M1 polarization. The levels of *miR-155*, inflammatory factors and chemokines were detected by qRT-PCR or ELISA. CD86, iNOS, SOCS3, JAK2, p-JAK2, STAT3 and p-STAT3 proteins expressions in macrophages were analyzed by western blotting. Conditioned medium transfer systems were designed to unite M1 macrophages with H/R cardiomyocytes, and cell apoptosis was detected by TUNEL staining, western blotting or immunohistochemistry.

**Results:**

SL reduced apoptosis, diminished CK and LDH levels, raised SOD concentration and decreased infarct size in the MI/R model. Meanwhile, SL decreased *miR-155* level, inhibited M1 macrophage polarization and inflammation. Furthermore, SL promoted SOCS3 expression and blocked JAK2/STAT3 pathway *in vitro*.

**Conclusions:**

SL may be a promising TCM candidate for MI/RI. The underlying mechanisms could be associated with inhibition of M1 macrophage polarization via down-regulating *miR-155*.

## Introduction

Myocardial ischaemia (MI) is associated with high morbidity and mortality. Timely reperfusion therapies, such as percutaneous coronary intervention and thrombolysis, are effective treatments to reduce ischaemic injury (Ibáñez et al. 2015). However, myocardial ischaemia reperfusion (MI/R) can also lead to further damage to myocardial tissue, myocardial ischaemia reperfusion injury (MI/RI), which increases initial infarct size (Toldo et al. 2018). Recent studies have suggested that the ischaemic cardiomyocytes can initiate activation of the innate immune system and induce acute inflammatory response featured with leukocyte infiltration in the infarcted heart at a background of MI/RI (Pluijmert et al. 2021). In addition, pharmacological inhibition of inflammation alleviated MI/RI *in vivo* and *in vitro*. Macrophages are the dominant innate immune cells that regulate the progression and resolution of inflammation. Functionally, macrophages can be divided into two typical subgroups: pro-inflammatory macrophages (M1) and anti-inflammatory macrophages (M2) (Hu et al. 2021). M1 macrophages express proinflammatory cytokines and proteolytic enzymes, which are used to clear cellular debris in the myocardial microenvironment. Nevertheless, the prolonged presence of M1 macrophages lengthens the pro-inflammatory state, degrades the extracellular matrix excessively and then induces cell death, which results in enlargement of infarct size and unexpected obstacles to heart repair (Ge et al. 2021).

MicroRNAs (miRNAs/miRs) are endogenous, noncoding RNAs with 19-24 nucleotides that are involved in cell proliferation, differentiation, apoptosis and metastasis. *miR-155*, as a well-known immunomodulatory miRNA, is mostly expressed in macrophages, monocytes and neutrophils (Mashima 2015). It has been reported that *miR-155* has a significant effect on cardiovascular diseases, such as myocardial ischaemia-reperfusion injury, acute myocardial infarction, atherosclerosis, heart failure and diabetic heart (Faraoni et al. 2009). the *miR-155* level was significantly raised in rats with MI/RI and *miR-155* knockout can ameliorate myocardial infarct and restrain cardiomyocyte apoptosis induced by ischaemia reperfusion (Guo et al. 2019). Furthermore, the increased expression of *miR-155* leads to M1 macrophage polarization and inflammatory responses. Suppressor of cytokine signalling 3 (SOCS3), one of the targets of *miR-155.* *miR-155* negatively regulates SOCS3 gene expression by binding to the 3- UTR of SOCS3 mRNAs and then suppresses the expression of the target protein SOCS3 (Henao Agudelo et al. 2017).

Traditional Chinese Medicine (TCM) has been used in diagnosing and treating diseases or disorders for thousands of years and is widely used in cardiovascular disease based on clinical experience. Shenlian extract (SL) is a combination of *Salvia miltiorrhiza* Bge. (Labiatae) and *Andrographis paniculata* (Burm. F.) Wall. Ex Nees (Acanthaceae) extracts. According to TCM theory, *S. miltiorrhiza* promotes blood circulation and removes blood stasis, *A. paniculata* has an impact on clearing endogenous heat toxins (Zhang et al. 2018; Li et al. 2021). Our previous study showed that SL stabilized atherosclerotic plaques in the atherosclerosis model constructed by ApoE^−/−^ mice and limited the crosstalk between macrophage and smooth muscle cells by down-regulate TGF-β expression (Liu et al. 2021). Meanwhile, SL improved myocardial ischaemic injury and regulated the immune system by reducing inflammatory cytokines and modulating the NF-kB pathway (Guo et al. 2020). Whether SL alleviates myocardial ischaemia-reperfusion injury has not been reported. In this study, we concentrate on investigating the effect of SL on MI/RI and the underlying mechanism. The research may be conducive to the potential application of SL in the clinical therapy of MI/RI.

## Methods and materials

### Reagents

*Salvia miltiorrhiza* and *A. paniculate* were purchased from Beijing Tongrentang and taxonomic authenticity was identified by Prof. Xi-rong He. Lipopolysaccharide (Cat. No: L2880-100MG), dimethyl sulfoxide (DMSO) (Cat. No: 276855) and methylthiazolyl tetrazolium (TTC) (Cat. No: CT01) were bought from Sigma. Foetal bovine serum (Cat. No: 10099141) and 0.25% Trypsin EDTA (Cat. No: 25300120) were obtained from Gibco. RIPA buffer (Cat. No: R0010) was purchased from Solarbio. Phosphatase inhibitor (Cat. No: 0490837001) and phenylmethylsulfonyl fluoride (Cat. No: P0100) were bought from Roche. p-STAT3 (Cat. No: 9415), STAT3 (Cat. No: 9139), cleaved caspase-9 (Cat. No: 9507S) and cleaved caspase-3 (Cat. No: 9661 T) antibodies were provided by CST. p-JAK2 (Cat. No: YP0785), JAK2 (Cat. No: YT2426) antibody was purchased from Immunoway. CD86 (Cat. No: A1199) antibody was bought from ABclonal. SOCS3 (Cat. No: A00274-2) antibody was purchased from BOSTER. iNOS (Cat. No: 18985-1-AP) antibody was obtained from Proteintech. Mouse TNF-α (Cat. No: 1217202), IL-1β (Cat. No:1210122), IL-6 (Cat. No: 1210602), IL-10 (Cat. No: 1211002) pre-coated ELISA kits were all purchased from Dakewe Biological Technology Ltd. RevertAid First Strand cDNA Synthesis Kit (Cat. No: 00984912) was bought from Thermoscientific. BCA (Cat. No: P0009) protein assay kit and SDS-PAGE (Cat. No: vP0009) were provided by Beyotime Biotechnology. SDS PAGE Running Buffer (Cat. No: vB1005) was bought from Applygen. Skim Milk (Cat. No: 3106120) was purchased from BD Biosciences. Bax (Cat. No: ZS74800), Bcl2 (Cat. No: ZS73820) and antibodies for immunohistochemistry were bought from ZSGB-BIO. SABC-AP (rabbit IgG) kit (Cat. No: SA1052) was obtained from BOSTER. A specific CY3-labelled *miR-155* probe (Cat. No: RX040864) was purchased from Servicebio. Creatine kinase (CK) (Cat. No: A032-1-1), lactate dehydrogenase (LDH) (Cat. No: A020-1-2) and superoxide dismutase (SOD) (Cat. No: A001-3-1) kits were bought from Nanjing Jiangcheng. PerfectStart^®^ Green qPCR SuperMix (Cat. No: AQ601) was purchased from Transgen.

### Extraction of herbs

The SL extract is composed of *S. miltiorrhiza* and *A. paniculata* extracts at a ratio of 5:3. *Salvia miltiorrhiza* extract consisted of two components, one was soaked in ethanol and extracted twice by refluxing for 2 h, and the filtrates were combined and concentrated under reduced pressure and below 60 °C, the centrifugal precipitate was the lipo-soluble extract. The second component was prepared by soaking in ethanol, after concentration and centrifugation, the supernatant was purified using macroporous resin (SP825). The *A. paniculata* extract was prepared by dilute ethanol soaking and purified by a macroporous resin (SP825). The extraction rate of the water-soluble partial extract of *S. miltiorrhiza* was 2.27% and that of the fat-soluble extract was 1.31%. The extraction rate of *A. paniculata* was 2.11%. In this study, Tanshinone IIA was detected from the lipo-soluble extract of *S. miltiorrhiza*, salvianolic acid B was analyzed from the water-soluble extract of *S. miltiorrhiza*, and andrographolide was tested from the extract of *A. paniculata*. The components from SL were tanshinone IIA (3%), salvianolic acid B (38%), and andrographolide (20%) and were detected by HPLC (Guo et al. 2020). During the *in vitro* experiment, the mixed extracts were dissolved in DMSO to prepare mother liquor, and then stored at −20 °C at 50 mg/mL.

### Animals

The study protocol was approved by the Experimental Animal Ethical Committee of the Institute of Chinese Materia Medica within the China Academy of Chinese Medical Science (2021B136).

Adult C57BL/6J mice 6–8 weeks old (weighted 20–25 g) were purchased from the Experimental Animal Centre of Military Medical Science Academy. The animals were housed in a quiet environment at 25 ± 2 °C and 55 ± 10% humidity, under a 12 h light/dark cycle.

### MI/R model and drug treatments

After 1 week of adaptation, all mice were divided into 6 groups (*n* = 10). Different groups adopted different treatment plans after grouping the mice. The positive control of the chemotherapy group was given 2.3 mg/kg atorvastatin (ATO) dissolved in 1% CMC-Na. The mice in the sham and model groups were given 1% CMC-Na only. The SL groups were given respectively three dosages of 90, 180 and 360 mg/kg SL (equivalent to 1-, 2- and 4-times of the clinical dose) dissolved in 1% CMC-Na. All mice were orally administered from day 1 after grouping, then every day for 8 days. On day 7 after treatment, the mice underwent surgery to induce MI/R model, briefly, after anaesthesia with an injection of avertin (16.5 mL/kg) into the abdominal cavity, mice were orally intubated and fixed using a small animal respirator. An incision was made from the third to fourth ribs to expose the heart. Hearts were then exposed through the left lateral thoracotomy. The left anterior descending (LAD) coronary artery was visualized and ligated with a 7-0 suture line. The suture was loosened after occlusion for 2 h, which was followed by 24 h reperfusion of LAD.

### TTC staining

After mice were sacrificed by anaesthesia, the hearts were taken and frozen for 20 min. Tissue sections under the ligation point were sliced, and then slices were placed in 2% TTC dye for 15 min at 37 °C. Next, the redundant staining solution was removed, and slices were washed three times with PBS and photographed for observation immediately.

### Contents determination of CK, LDH and SOD

CK, LDH and SOD in mice serum or heart tissue contents analysis was carried out according to the kit instructions.

### Mouse peritoneal macrophages (pMACs) culture

Peritoneal macrophages were elicited by intraperitoneal injection of 2 mL 3% sterile starch solution, 3 days later, the animals were sacrificed, peritoneal lavage with 10 mL PBS and was rotated 225 *g* for 5 min to collect macrophages. The peritoneal macrophages were cultured in an RPMI-1640 cell culture medium containing 10% FBS, 1% penicillin and streptomycin. After incubation at 37 °C and 5% CO_2_ for 24 h, non-adherent cells were removed to obtain pure peritoneal macrophages.

### Induction of M1 macrophages and drug treatment in vitro

Peritoneal macrophages were divided into 5 groups (*n* = 3). The pMACs in the control group were given 1% DMSO. The pMACs in the model group were stimulated with LPS (600 ng/mL) supplementing 1% DMSO. The pMACs in the SL groups (named SL-5, SL-10 and SL-20, respectively) were treated with 5, 10 or 20 μg/mL SL containing LPS (600 ng/mL) and 1% DMSO. Then pMACs were cultured for 24 h in the medium supplementing 10% FBS, 1% penicillin and streptomycin at 37 °C and 5% CO_2_.

### Cell culture

H9C2 rat cardiomyoblast cell line was purchased from ATCC and cultured in DMEM supplemented with 10% FBS, 1% streptomycin and penicillin. Cells were maintained at 37 °C in a humidified incubator with 5% CO_2_.

### Hypoxia reoxygenation (H/R) model *in vitro*

When cell density reached 90%, cells were exposed to hypoxic conditions (oxygen deprivation, 94% N_2_, 5% CO_2,_ and 1% O_2_) at 37 °C for 6 h in a culture medium with lower glucose and serum-free. After hypoxia, the medium was removed and the cells were placed in a reoxygenation environment (reoxygenation, 21% O_2_ and 5% CO_2_) at 37 °C for 5 h in a normal medium. The control group cells without hypoxia treatment were maintained in normoxic conditions.

### Conditioned medium transfer systems

To demonstrate that SL had an effect on H9C2 cells under H/R through M1 macrophage polarization, we established conditioned transfer model 1. As shown in Figure 5(A), peritoneal macrophages were seeded and then stimulated with LPS (600 ng/mL) and treated with SL (5, 10, 20 μg/mL) for 24 h, the medium (LPS and SL-containing) was collected as macrophage-conditioned media 1(CM1), and then CM1 was diluted to one third (CM1/3) of its original concentration and applied for H/R H9C2 cells.

To eliminate the direct influence of SL on H9C2, conditioned medium transfer model 2 was established. In Figure 5(D), peritoneal macrophages were seeded and then stimulated with LPS (600 ng/mL) and treated with SL (5, 10 and 20 μg/mL) for 24 h. Then, the medium (LPS and SL-containing) was removed and fresh medium was added to pMACs for 24 h and the medium (LPS and SL-free) was collected as macrophage-conditioned media (CM2). Finally, CM2 was diluted to one-third (CM2/3) of its original concentration and applied to H/R H9C2 cells.

### Western blotting analysis

Western blotting analyses were done to measure the expression of the p-STAT3, STAT3, p-JAK2, JAK2, CD86, iNOS, SOCS3, cleaved caspase-3, cleaved caspase-3, caspase-3, Bcl2 and Bax. For the preparation of protein extracts, cell pellets were homogenized by RIPA buffer supplemented with aprotinin (10 mg/mL) and leupeptin (10 mg/mL) for 30 min on ice. The mixture was rotated at 12,000 *g* for 15 min to remove the insoluble fragments. the supernatant was collected to obtain the total protein. After being subjected to quantification using the BCA assay, the protein samples were separated by 10% SDS-PAGE and transferred to PVDF membranes. Membranes were blocked for 3 h with 5% skim milk or 5% BSA and then incubated with primary antibodies at 4 °C overnight. After washing, appropriate secondary antibodies were used to the membranes for 1 h at room temperature. The blots were developed by enhanced chemiluminescence detection reagents. Quantification of the gray intensity of the protein band performed by using ImageJ and normalized to the gray intensity of GAPDH.

### Quantitative real-time reverse transcription polymerase chain reaction (qRT-PCR)

*miR-155* expression was detected by qRT-PCR. Briefly, total RNA from tissue or cell pellets was extracted with Trizol Reagent. cDNA was synthetized by a Transcriptor First Strand cDNA Synthesis Kit. Then cDNA templates were amplified using RNA primers and PerfectStart^®^ Green qPCR SuperMix according to the manufacturer’s instructions. The following mouse primers (forward and reverse, respectively) were used: 5′-GGGCTTAATGCTAATTGTGAT-3′ and 5′-CAGTGCGTGTCGTGGAGT-3′ for *miR-155*; 5′-CTCGCTTCGGCAGCACA-3′ and 5′-AACGTTCACGAATTTGCGT-3′ for U6. Expression was calculated using the comparative-threshold cycle method and normalized to the expression of U6 mRNA. Genes expression of CCR2, CCL2, CXCL1, IL-6, TNF-α, CXCR1 were detected by RT-PCR. Similarly, the cDNA templates were amplified using RNA primers and 2 × GoldStar Best MasterMix according to the manufacturer’s protocol after total RNA had been extracted and cDNA synthetized. The following mouse primers (forward and reverse, respectively) were used: 5′-CTGAACGGGAAGCTCACTGG-3′ and 5′-TCCGATGCCTGCTTCACTAC-3′ for GAPDH; 5′-CCACTCACCTGCTGCTACTCATTC-3′ and 5′-CTGCTGCTGGTGATCCTCTTGTAG-3′ for CCL2; 5′-GCTCATCTTTGCCATCATGATT-3′ and 5′-TCATTCCAAGAGTCTCTGTCAC-3′ for CCR2; 5′-CCGGTACCGGAGGCCGCGCTCGCGGG-3′ and 5′-AACAGATCTCGCGCAGCACCAAACTGCC-3′ for SOCS3. RNA expression was measured through agarose-gel electrophoresis and results were visualized and photographed using an ultraviolet transilluminator. We quantified the gray intensity of the DNA band using ImageJ and normalized it to the gray intensity of GAPDH.

### Enzyme-linked immunosorbent assay (ELISA) analysis

Primary macrophages were treated for 24 h and cells supernatant was collected and stored at −20 °C. The concentrations of IL-1β, IL-6, TNF-α and IL-10 in the supernatant were determined by the corresponding mouse ELISAs according to manufacturer instructions.

### Histological assessments

Mouse heart tissue was harvested and fixed with 4% paraformaldehyde, dehydrated, embedded in paraffin, and transversely sectioned into 5 µm pieces, which were baked at 60 °C for 12 h and dehydrated with graded ethanol series for histological assessments. In the immunofluorescence assay, the tissues were heated in citrate buffer (pH = 7.4) for 15 min to recover antigens. After being blocked with 5% BSA in Tris-buffered saline for 30 min at 37 °C and sections were incubated with antibody mixes at 4 °C overnight, after that, slides were incubated with the secondary antibodies (1:200 dilution each) for 1 h, and then DAPI was used as counterstain. Finally, Fluorescence images were acquired using a Nikon inverted fluorescence microscope. Primary antibodies used for immunofluorescence staining are followed: anti-F4/80 rat monoclonal (1:100 dilution), anti-CD86 rabbit polyclonal (1:100 dilution), anti-iNOS rabbit polyclonal (1:100 dilution).

For immunohistochemical staining, first, the sections were covered with 3% H_2_O_2_ for 15 min at room temperature and antigen retrieval was carried out at 95 °C for 15 min in a citrate buffer (pH = 7.4). The sections were incubated with primary antibodies at 4 °C overnight after being blocked with 5% BSA. Then, the slides were incubated with biocatalytic secondary antibody (1:200 dilution) for 1 h at room temperature and streptavidin-horseradish peroxidase for another 30 min, positive signalling was magnified by adding a DAB-H_2_O_2_ solution. Finally, the slides were counterstained with haematoxylin for 3 min and fixed in neutral balsam after dehydration with ethanol. Primary antibodies used for immunohistochemistry staining are followed: mouse anti-Bax (1:100 dilution) and mouse anti-Bcl2 (1:100 dilution).

Apoptosis of mouse heart tissue and H9C2 cells were detected by TUNEL assay using terminal deoxynucleotidyl transferase dUTP nick end labelling staining using the TUNEL cell death detection kit. H9C2 cells were seeded in 24 well plates, at 4 × 10^4^ cells/well. 5 μm thick sections of paraffin-embedded tissue were deparaffinized and hydrated in graded alcohol solutions. After slides and cells were incubated with 0.1% Triton X-100 for 10 min at 37 °C, TdT Equilibrium solution was added for 30 min, next, labelling working dye composed of TDT Equilibration, labelling solution and TDT Enzyme was employed to banding the apoptotic cells for 1 h at 37 °C. DAPI was used to stain the nuclei. Finally, TUNEL-positive nuclei were detected at 570–620 nm by fluorescence microscopy.

### Immunofluorescence in situ hybridization

Immunofluorescence *in situ* hybridization was performed as described previously (Wang et al. 2019). Slices were dewaxed in reduced concentration ethanol. Primary macrophages were seeded in 24 well plates and were incubated with 0.1% Triton X-100 for 10 min at 37 °C. And then added prehybridization buffer on the cells and slices at 37 °C for 1 h, next the tissue and cells were incubated using mixes containing CY3-labelled *miR-155* probe (50 nM) and anti-F4/80 rat monoclonal antibody or mixes containing CY3-labelled *miR-155* probe (50 nM) and anti-CD86 rabbit polyclonal antibody at 4 °C overnight, Finally, cells and slices were incubated with the secondary antibodies (1:200 dilution) for 1 h and DAPI was employed for nuclear staining.

### Statistical analysis

Results are expressed as the mean ± SD of three or more independent experimental results. Statistical analysis was performed by Student’s *t-*test or one-way analysis of variance followed by SPSS 17.0 software using the one-way ANOVA. *p* < 0.05 was considered statistically significant.

## Results

### SL improved myocardial ischaemia reperfusion injury in vivo

**Figure 1.**
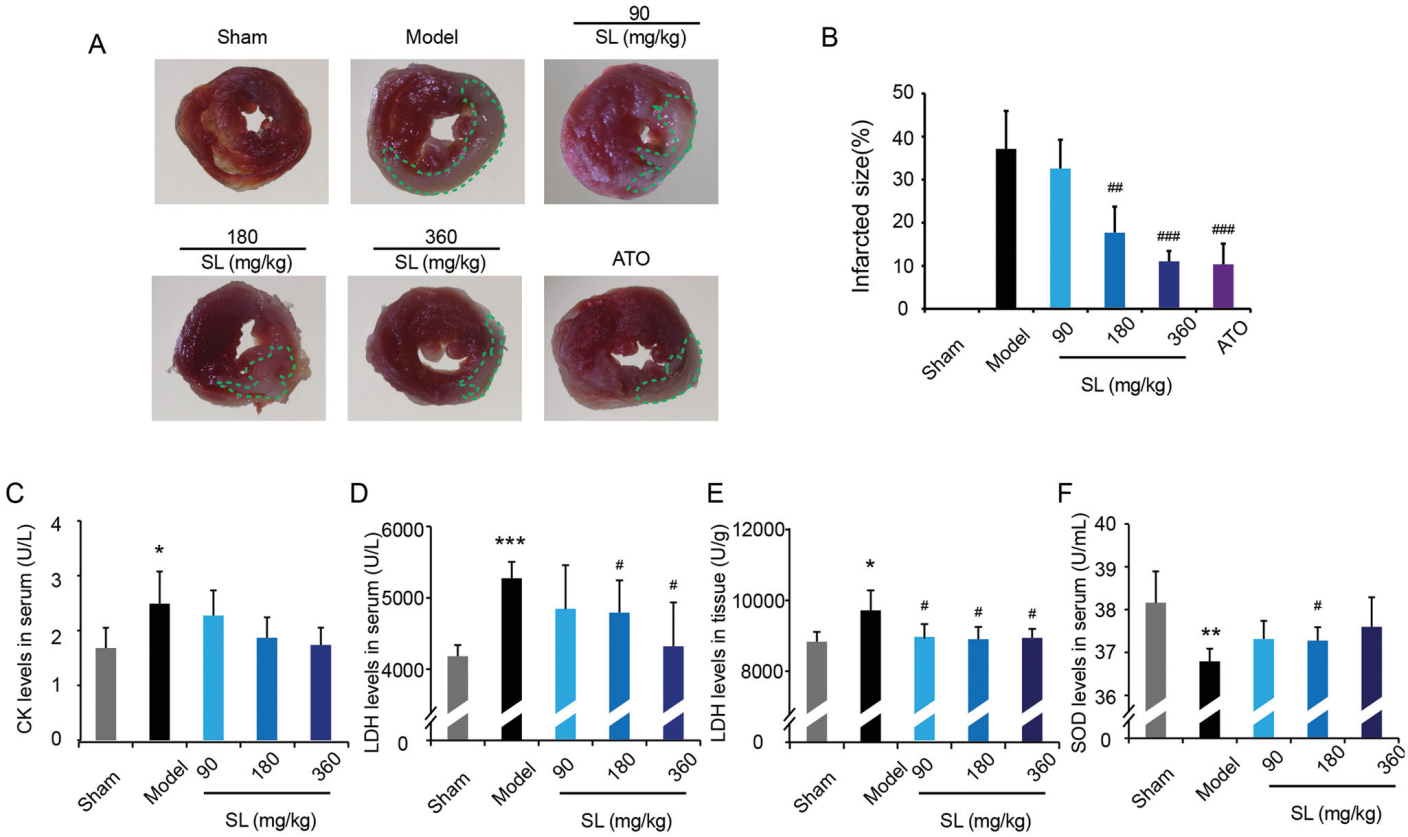
The effect of SL on MI/RI. (A, B) TTC staining and quantitative assay in mice heart tissue. (C–F) CK, LDH, SOD levels in serum or heart tissue. Results were presented as mean ± SD. *p < 0.05, **p < 0.01, ***p < 0.001 vs. sham group, ^#^*p* < 0.05, ^##^*p* < 0.01, ^###^*p* < 0.001 vs. model group, *n* = 6.

We evaluated the effect of SL on myocardial ischaemia-reperfusion injury *in vivo* by TTC staining, results revealed that a remarkable increase in infarcted size was observed in the model group compared with the sham group, and infarcted size in SL (180 mg/kg), SL (360 mg/kg) and ATO groups were decreased sharply in comparison to the model group (Figure 1(A,B); *p* < 0.01 or *p* < 0.001). Additionally, data suggested that compared with those in the sham group, the levels of CK and LDH in serum or tissues were increased significantly and SOD activity declined dramatically in the model group (Figure 1(C–F); *p* < 0.05, *p* < 0.01 or *p* < 0.001). In SL groups, the levels of CK and LDH in serum or tissue were lower and activity of SOD was higher than those in the model group, and in SL (180 mg/kg) group, the levels of LDH were lower notably and SOD concentration was significantly higher than the model group (Figure 1(C–F); *p* < 0.05).

### SL diminished apoptosis induced by MI/RI

**Figure 2.**
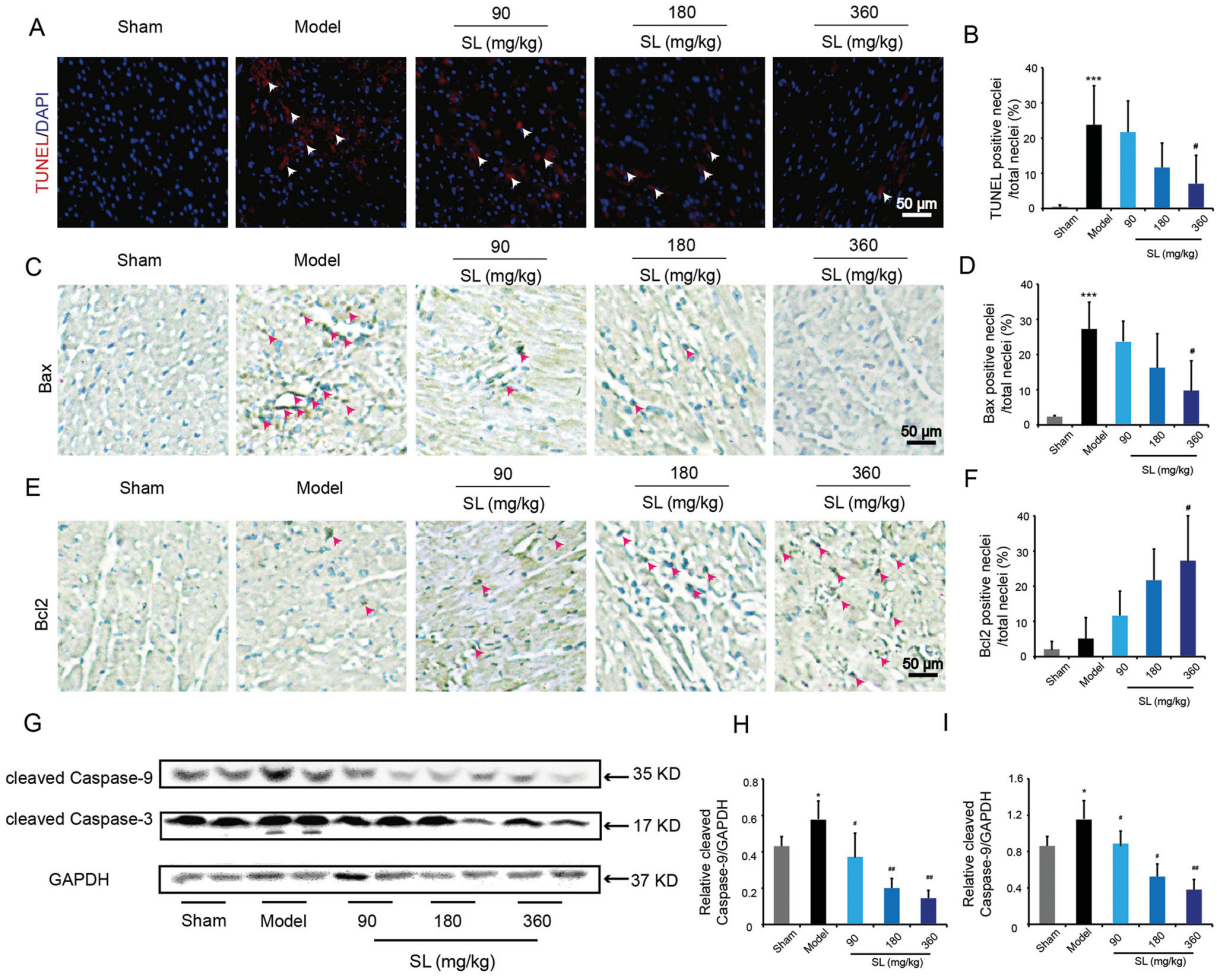
The effect of SL on apoptosis in mice MI/RI model. (A, B) TUNEL immunofluorescence staining and quantitative assay in heart tissue, scale bar = 50 μm. (C–F) Bcl2, Bax proteins expression and quantitative analysis in tissue by immunohistochemistry, scale bar = 50 μm. (G–I) Cleaved caspase-3, cleaved caspase-9 proteins expression and quantitative analysis in tissue by western blotting. Results were presented as mean ± SD. ***p < 0.001 vs. sham group, ^#^*p* < 0.05 vs. model group, *n* = 3.

Furthermore, we examined the effects of SL on apoptosis in MI/R mice by TUNEL staining. More positive signals of TUNEL were found in the model group and less positive signals of TUNEL were found in SL (360 mg/kg) group (Figure 2(A,B); *p* < 0.05). Immunohistochemistry results also showed that compared with the sham group, the levels of Bax increased significantly and expression of Bcl2 diminished notably in the model group. However, compared with the model group, the levels of Bax decreased obviously and expression of Bcl2 raised remarkably in SL (360 mg/kg) groups (Figure 2(C–F); *p* < 0.05). Continuously, western blotting results also suggested that the levels of cleaved caspase-3 and cleaved caspase-9 increased significantly in the model group and decreased dramatically in SL groups (Figure 2(G–I); *p* < 0.05 or *p* < 0.01).

### SL suppressed M1 macrophage polarization in MI/R model

**Figure 3.**
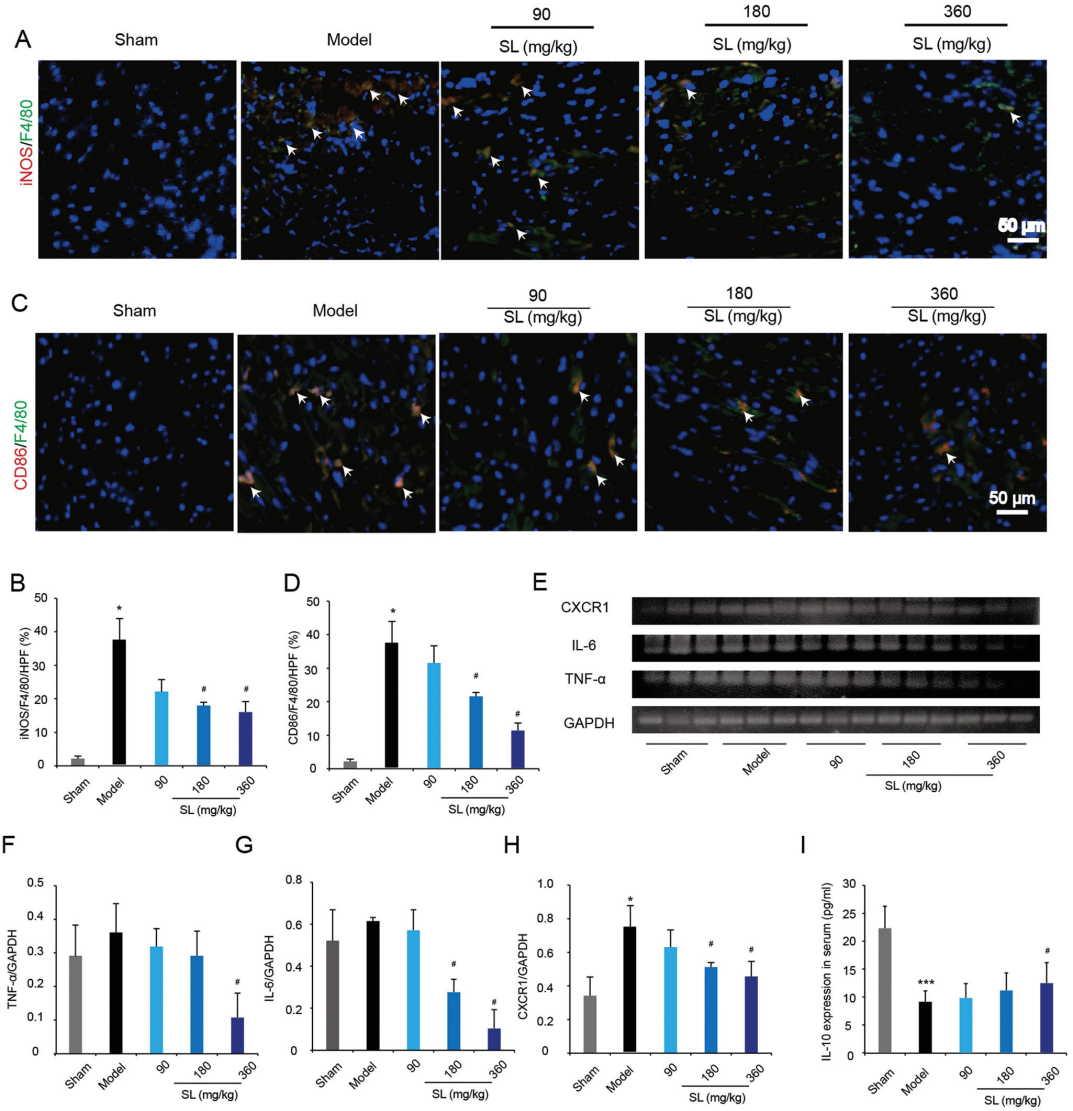
The effect of SL on M1 macrophages polarization in MI/R mice. (A, B) Immunofluorescence double staining of iNOS/F4/80 and quantitative analysis in mice heart tissue, scale bar = 50 μm. (C, D) Immunofluorescence double staining of CD86/F4/80 and quantitative analysis in mice heart tissue, scale bar = 50 μm. (E–H) IL-6, TNF-α, CXCR1 genes expression in tissue by RT-PCR. (I) IL-10 level in serum by ELISA. Results were presented as mean ± SD. *p < 0.005, ***p < 0.001 vs. sham group, ^#^*p* < 0.05 vs. model group, *n* = 3.

Subsequently, we tested the effect of SL on macrophage polarization by double immunofluorescence staining *in vivo*. F4/80 is a macrophage surface maker, and CD86 and iNOS are M1 macrophages signals, thus, double immunofluorescence staining of iNOS/F4/80 and CD86/F4/80 was used to bind M1 macrophages. Our results showed that iNOS/F4/80 and CD86/F4/80 positive signals increased significantly in the model group compared with those in the sham group, in SL (180 mg/kg) and SL (360 mg/kg) groups, F4/80/iNOS and F4/80/CD86 positive markers decreased notably compared with that in the model group (Figure 3(A–D); *p* < 0.05).

Based on the effect of SL on M1 macrophage polarization, we detected levels of inflammatory cytokines and chemokines in mice. qRT-PCR and ELISA results suggested that in the model group, IL-6, TNF-α, CXCR1increased and anti-inflammatory factors IL-10 decreased significantly compared with that in the sham group. However, in SL (360 mg/kg) group, IL-6, TNF-α, CXCR1 levels were notably lower and IL-10 concentration was obviously higher than model group (Figure 3(E–I); *p* < 0.05).

### SL reduced *miR-155* expression in MI/R model

**Figure 4.**
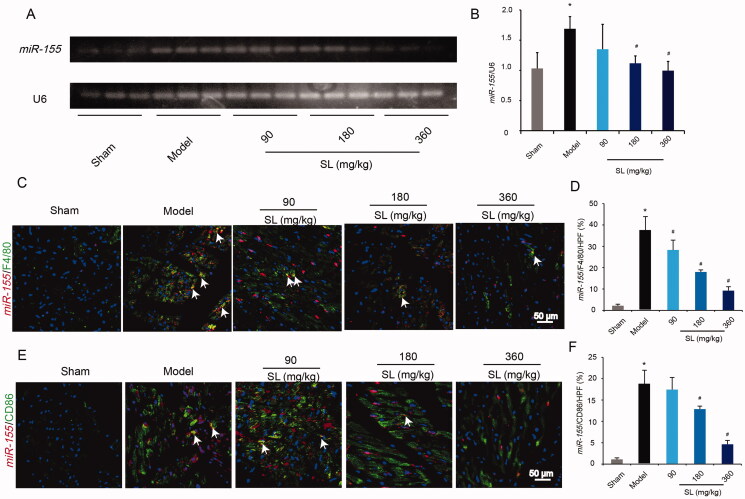
The effect of SL on *miR-155* expression *in vivo*. (A, B) *miR-155* level and quantitative analysis in mice heart tissue by qRT-PCR. (C, D) *miR-155*/F4/80 positive expression and quantitative analysis in mice heart tissue, scale bar = 50 μm. (D) *miR-155*/CD86 positive expression and quantitative analysis in mice heart tissue, scale bar = 50 μm. Results were presented as mean ± SD. *p < 0.005 vs. sham group, ^#^*p* < 0.05 vs. model group, *n* = 3.

A previous study reported that increasing the expression of *miR-155* resulted in M1 macrophage polarization (Pasca et al. 2020). To explore the potential mechanism concerning the inhibition effect of SL on M1 macrophage polarization, we examined the expression of *miR-155* in macrophages by qRT-PCR. As shown in Figure 3(A,B), gene expression of *miR-155* increased significantly in the model group by comparison to the sham group (*p* < 0.05). However, compared with that in model mice, expression of *miR-155* decreased notably in SL (180 mg/kg) and SL (360 mg/kg) groups (Figure 4(A,B); *p* < 0.05). *miR-155*/F4/80 double-staining results showed that compared to the sham group, *miR-155*/F4/80 positive makers in the model group raised evidently and *miR-155*/F4/80 positive expression diminished significantly in SL groups in comparison with the model group (Figure 4(C,D); *p* < 0.05). Simultaneously, *miR-155*/CD86 double-staining demonstrated that *miR-155*/CD86 positive expression in the model group was remarkably more than in the sham group, and *miR-155*/CD86 positive signals in SL (180 mg/kg) and SL (360 mg/kg) groups evidently were less than the model group (Figure 4(E,F); *p* < 0.05).

### SL reduced M1 macrophage conditioned media-induced cardiomyocyte apoptosis in H/R injury

**Figure 5.**
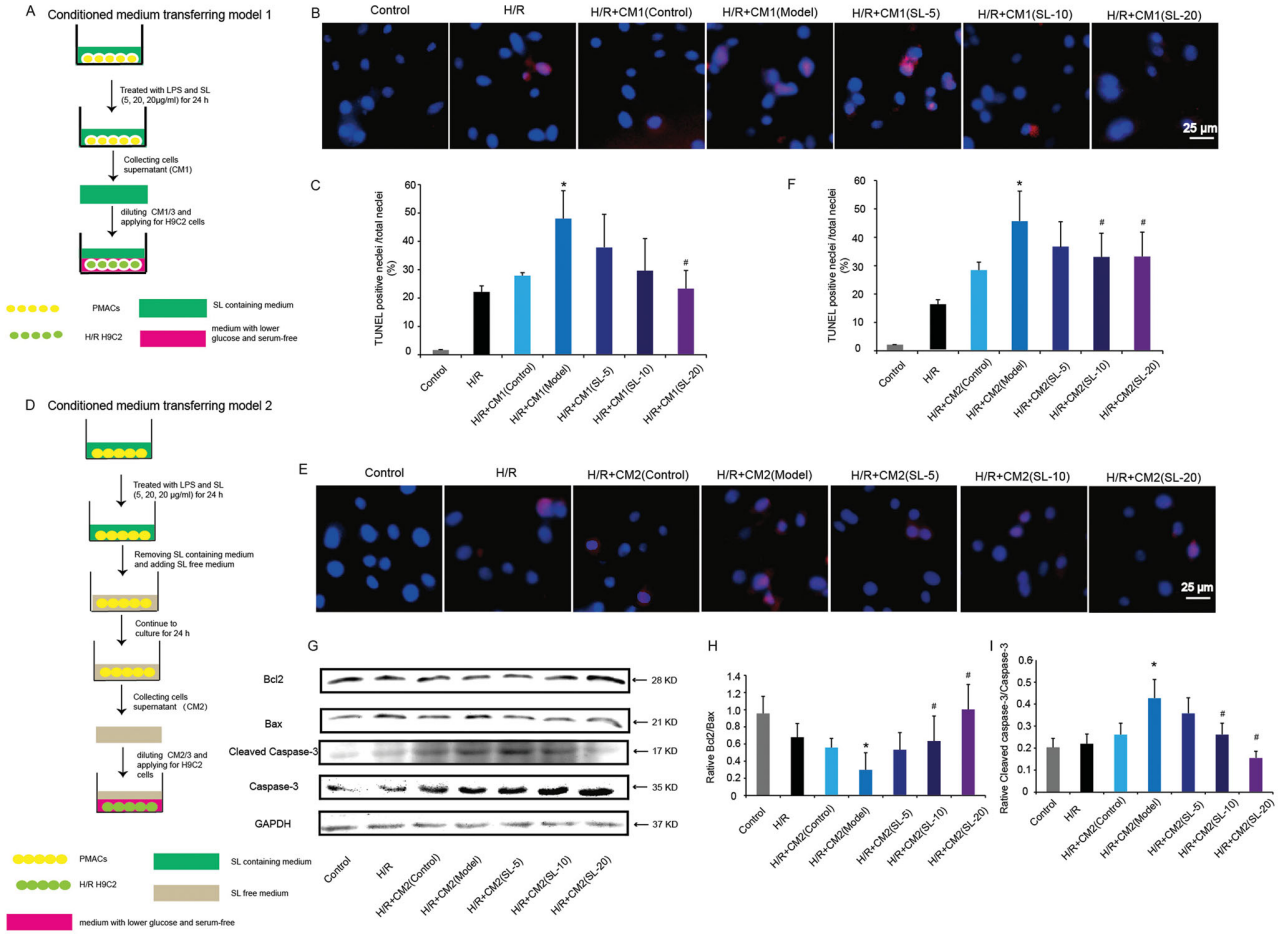
The effect of SL on H/R H9C2 induce by M1 macrophages. (A) The protocol of pMACs/H9C2 conditional medium transferring model 1. (B, C) TUNEL immunofluorescence staining and quantitative assay in conditional medium transferring model 1, scale bar = 25 μm. Results were presented as mean ± SD. *p < 0.05 vs. H/R + CM1(control) group, ^#^*p* < 0.05 vs. H/R + CM1(model) group, *n* = 3. (D) The protocol of pMACs/H9C2 conditional medium transferring model 2. (E, F) TUNEL immunofluorescence staining and quantitative assay in conditional medium transferring model 2, scale bar = 25 μm. (G–I) Bcl2, Bax, caspase-3, cleaved caspase-3 proteins expression and quantitative analysis in conditional medium transferring model 2 by western blotting. Results were presented as mean ± SD. *p < 0.05 vs. H/R + CM2 (control) group, ^#^*p* < 0.05 vs. H/R + CM2 (model) group, *n* = 3.

To investigate whether SL relieved M1 macrophage conditioned media-induced H/R cardiomyocyte damage, we designed the conditioned medium transfer models. In conditioned medium transfer model 1, TUNEL immunofluorescence results suggested that positive signals of H9C2 were increased notably in the H/R + CM1(model) group in comparison with the H/R + CM1(control) group, however, apoptotic cells were significantly decreased in H/R + CM1(SL-20) group compared with H/R + CM1(model) group (Figure 5(B,C), *p* < 0.05). Meanwhile. In the conditioned medium transfer model 2, apoptotic H9C2 was elevated in the H/R + CM2(model) group in relation to the H/R + CM2(control) group. Concurrently, the ratio of the positive cell declined notably in H/R + CM2(SL-10) and H/R + CM2(SL-20) groups compared with the H/R + CM2(model) group (Figure 5(D–F), *p* < 0.05). Besides, we tested proteins expression of Bcl2, Bax, caspase-3 and cleaved caspase-3 by western blotting in the conditioned medium transfer model 2. Our results showed that compared with the CM2(control) group, levels of cleaved caspase-3, Bax and ratio of cleaved caspase-3/caspase-3 increased and the ratio of Bcl2/Bax decreased in H/R + CM2(model) group. However, compared with the CM2 (model) group, levels of cleaved caspase-3, the ratio of cleaved caspase-3/Caspase-3 diminished, Bcl2 expression and the ratio of Bcl2/Bax raised in H/R + CM2 (SL-10) and H/R + CM2 (SL-20) groups (Figure 5(G–I), *p* < 0.05).

### SL inhibited M1 macrophages polarization mediated by LPS

**Figure 6.**
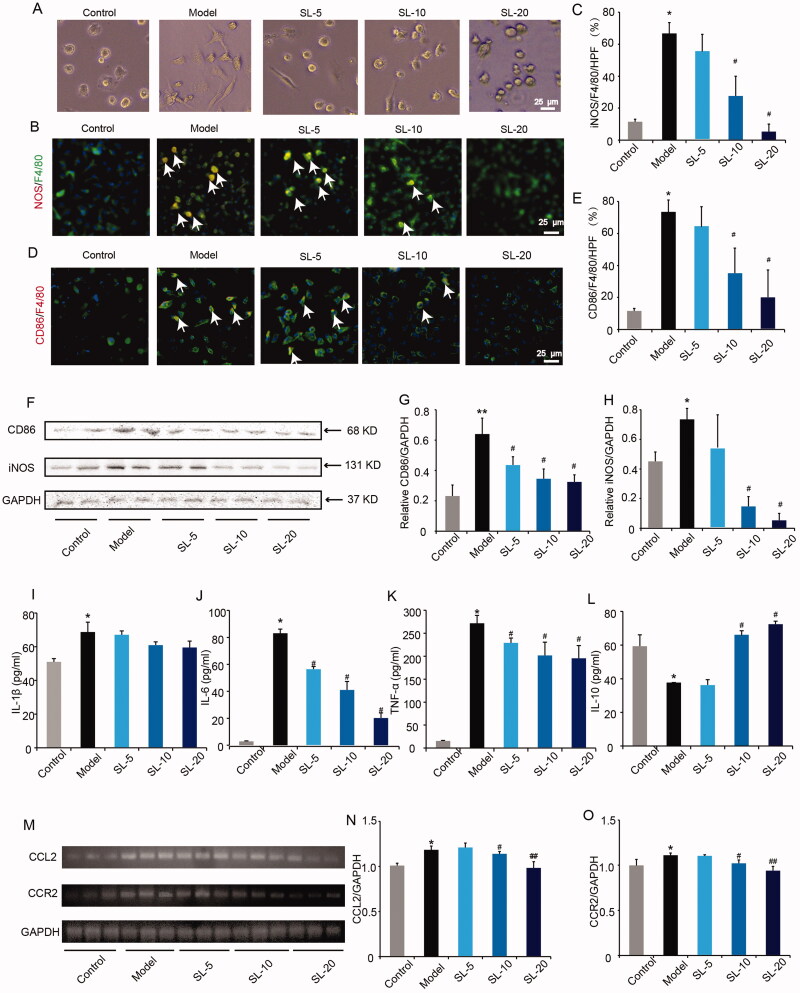
The effect of SL on M1 macrophages polarization. (A) The morphological changes of pMACs. scale bar = 25 μm. (B, C) Double immunofluorescence staining of iNOS/F4/80 and quantitative analysis in pMACs, scale bar = 25 μm. (D, E) Double immunofluorescence staining of CD86/F4/80 and quantitative analysis in pMACs, scale bar = 25 μm. (F–H) iNOS and CD86 proteins expression and quantitative results in pMACs. (I–L) Inflammatory factors IL-1β, IL-6, TNF-α, IL-10 levels in pMACs. (M–O) Chemokines CCL2, CCR2 expression in pMACs. Results were presented as mean ± SD. *p < 0.05, **p < 0.01 vs. control group, ^#^*p* < 0.05, ^##^*p* < 0.01 vs. model group, *n* = 3.

*In vitro*, we established the protocol to polarize pMACs into M1 macrophages via exposure to LPS (600 ng/mL). After 24 h of co-culture with LPS, the morphological changes of pMACs were observed, and LPS-treated pMACs showed obvious morphological abnormalities from the oval to the spindle. Besides, there were dendritic processes on the edge of cells in comparison with unstimulating pMACs. Conversely, when macrophages were incubated with LPS in the presence of SL (5, 10 or 20 μg/mL), the cells did not tally with the larger and more fusiform appearance characteristic of LPS-treated macrophages but more closely resemble macrophages cultured in medium alone which suggested that SL can restore M1 macrophages morphological changes induced by LPS (Figure 6(A)). Concurrently, we labelled M1 macrophages by double immunofluorescence staining of iNOS/F4/80 and CD86/F4/80. We discovered obviously more iNOS/F4/80 and CD86/F4/80 makers in the model group, whereas iNOS/F4/80 and CD86/F4/80 signals were reduced significantly in SL-10 and SL-20 groups (Figure 6(B–E); *p* < 0.05). Next, we detected the M1 macrophage marker proteins expression of CD86 and iNOS by western blotting. As shown in Figure 6(F–H), LPS at 600 ng/mL upregulated the expressions of CD86 and iNOS of macrophages significantly compared with the control group. And after treatment with SL, the expressions of CD86 and iNOS decreased remarkably (*p* < 0.05).

Concurrently, we tested the levels of inflammatory cytokines by ELISA and chemokines by qRT-PCR. In the model group, levels of proinflammatory factors IL-1β, IL-6 and TNF-α notably increased and expression of anti-inflammatory factors IL-10 decreased significantly compared with the control group (Figure 6(I–L); *p* < 0.05). Contrarily, in SL groups, levels of IL-6 and TNF-α were less and in SL-10 and SL-20 groups, IL-10 concentration was remarkably more than in the model group (Figure 6(I–L); *p* < 0.05). Similarly, genes expression of chemokines CCL2 and CCR2 raised markedly in LPS-treated macrophages in comparison with control macrophages, while in SL-10 and SL-20 groups, genes expression of CCL2 and CCR2 reduced notably compared with the model group (Figure 6(M–O); *p* < 0.05 or *p* < 0.01).

### SL down-regulated *miR-155* expression* in vitro*

**Figure 7.**
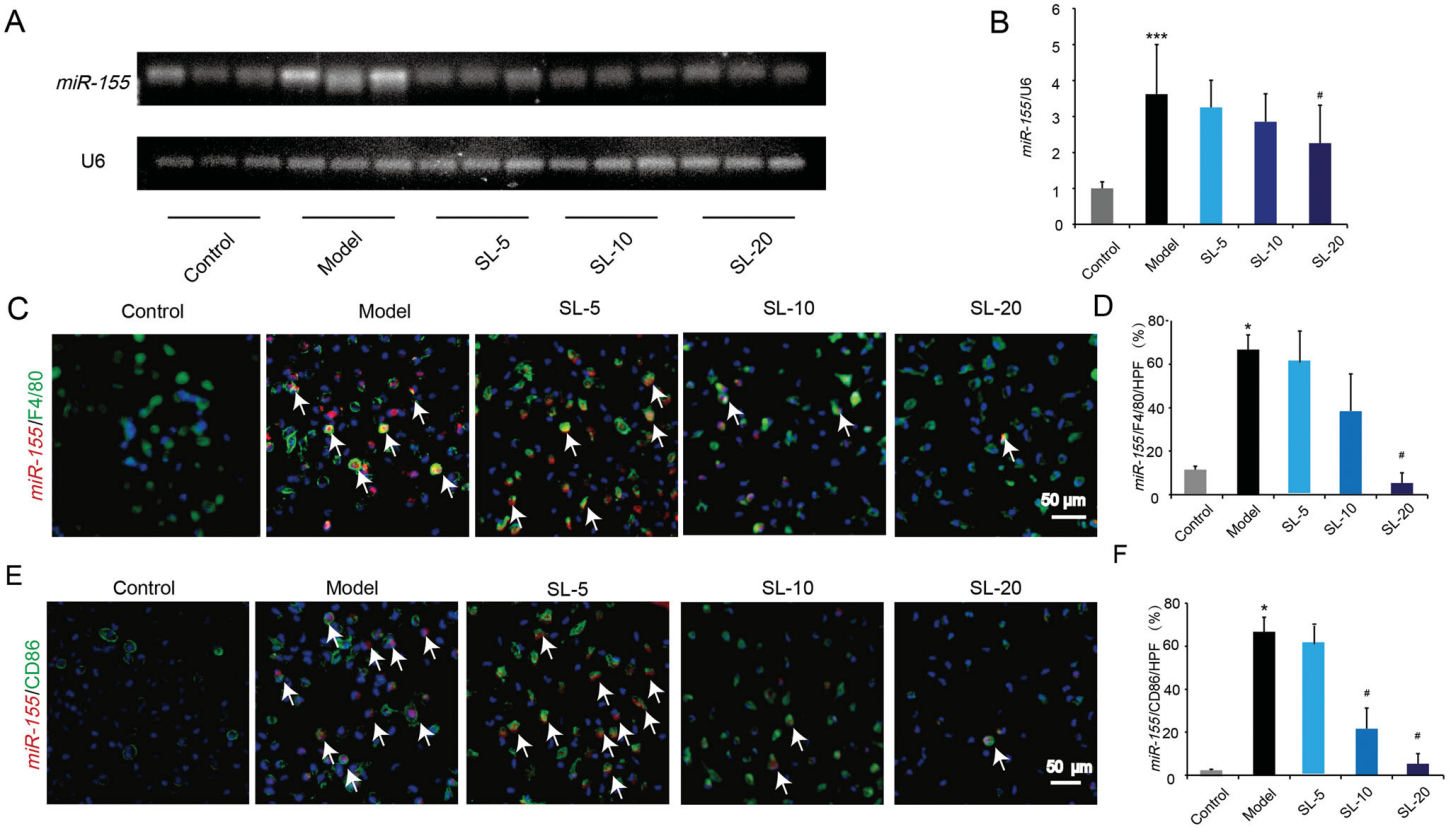
The effect of SL on *miR-155*. (A, B) *miR-155* level and quantitative analysis in pMACs by qRT-PCR. (C, D) *miR-155*/F4/80 positive expression in pMACs and quantitative analysis, scale bar = 50 μm. (E, F) *miR-155*/CD86 positive expression and quantitative analysis and quantitative analysis, scale bar = 50 μm. Results were presented as mean ± SD. *p < 0.05, ***p < 0.001 vs. control group, ^#^*p* < 0.05 vs. model group, *n* = 3.

Next, we examined the expression of *miR-155* *in vitro.* In Figure 7(A,B), gene expression of *miR-155* increased significantly in LPS-treated macrophages in comparison with control macrophages, and in co-treated LPS and SL macrophages gene, expression of *miR-155* reduced in a dose-dependent compared with LPS-treated macrophages (Figure 7(A,B), *p* < 0.05). Meanwhile, fluorescence *in situ* hybridization results showed that *miR-155*/F4/80 positive makers in the model group were more than that in the control group, and in the SL groups *miR-155*/F4/80 expressions were less than that in the model group (Figure 7(C,D), *p* < 0.05). Furthermore, fluorescence *in situ* hybridization of *miR-155*/CD86 results illustrated that *miR-155*/CD86 positive signals in the model group raised significantly compared with the control group, but in the SL groups, *miR-155*/CD86 expressions decreased notably compared with the model group (Figure 7(E,F), *p* < 0.05).

### SL up-regulated SOCS3 expression in vitro

**Figure 8.**
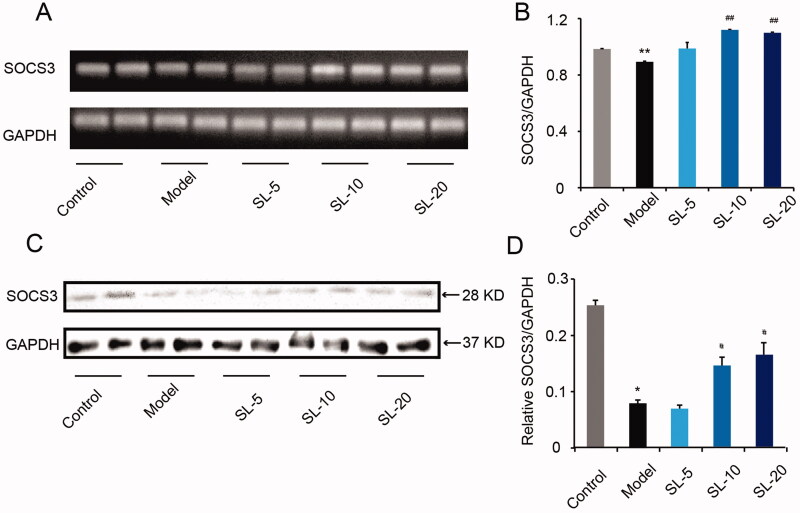
The effect of SL on SOCS3 expression. (A, B) SOCS3 gene expression and quantitative analysis by qRT-PCR. (C, D) SOCS3 proteins expression of and quantitative analysis in pMACs by western blotting. Results were presented as mean ± SD. *p < 0.05, **p < 0.01 vs. control group, ^#^*p* < 0.05, ^##^*p* < 0.01 vs. model group, *n* = 3.

SOCS3 is one of the targets of *miR-155* while SOCS3 deficiency induces M1 macrophage polarization and inflammatory (Qin et al. 2012). In this study, we tested SOCS3, as shown in Figure 8(A–D), gene and protein expression of SOCS3 notably decreased in LPS-stimulated macrophages compared with that in untreated macrophages, and in macrophages treated with LPS in the presence of SL (10 and 20 μg/mL) macrophages, gene and protein expression of SOCS3 increased remarkably in comparison with that in LPS-treated macrophages (*p* < 0.05).

### SL blocked JAK2/STAT3 signalling pathway *in vitro*

**Figure 9.**
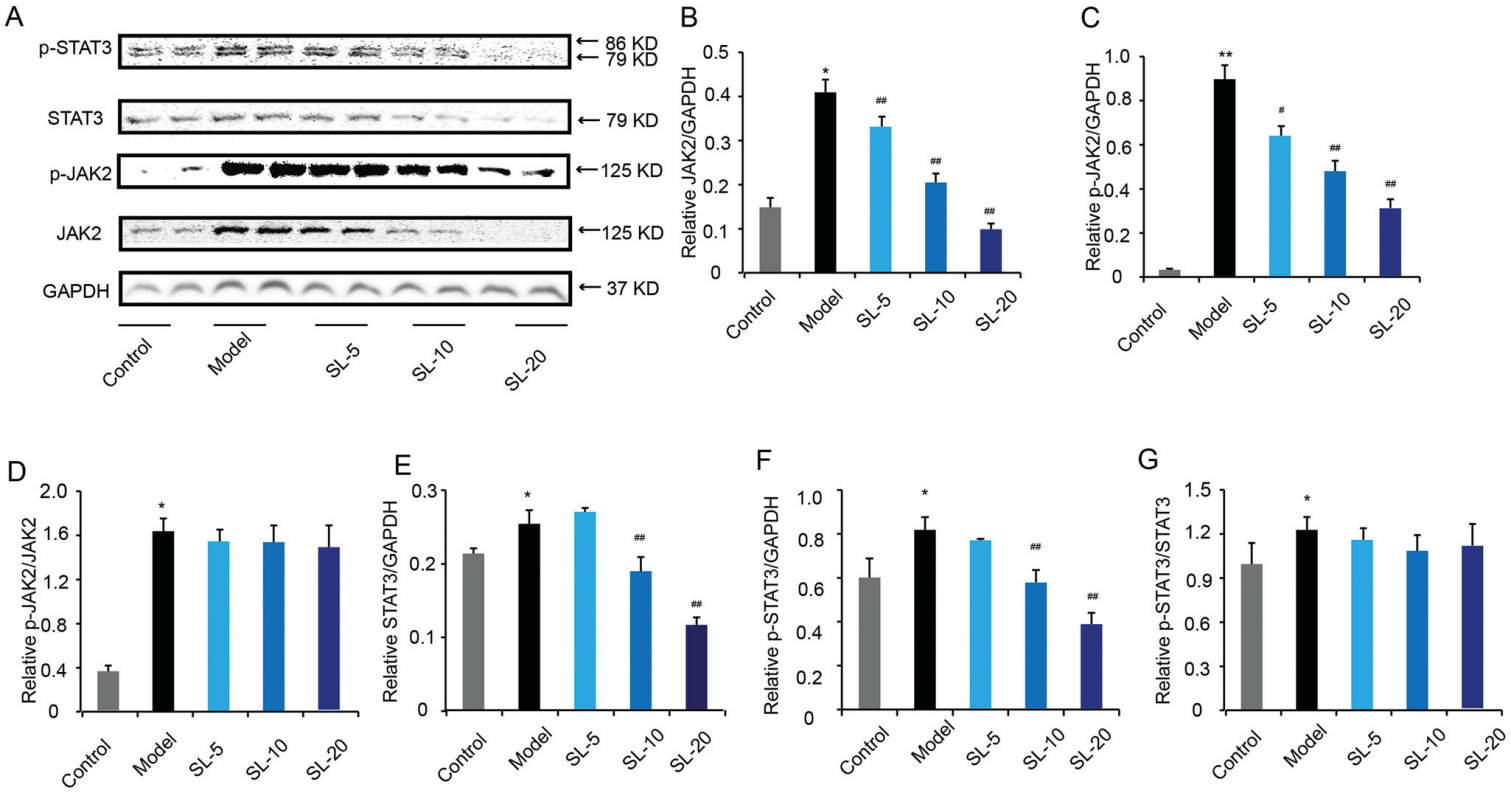
The effect of SL on JAK2/STAT3 pathway. (A–G) JAK2, STAT3, p-JAK2, p-STAT3 protein expression of and quantitative analysis in pMACs by western blotting. Results were presented as mean ± SD. *p < 0.05, **p < 0.01 vs. control group, ^#^*p* < 0.05, ^##^*p* < 0.01 vs. model group, *n* = 3.

JAK2/STAT3 pathway activation induces the inflammatory reaction (Chen et al. 2019). In this study, we tested JAK2, p-JAK2, STAT3 and p-STAT3 expressions by western blotting. In Figure 9(A–G), the expression of JAK2, STAT3, p-JAK2, p-STAT3 expressions and ratios of p-JAK2/JAK2, p-STAT3/STAT3 in the model group were higher than the control group. In SL-10 and SL-20 groups, JAK2, STAT3, p-JAK2 and p-STAT3 were lower than model group (*p* < 0.05), but in SL groups, ratios of p-JAK2/JAK2, p-STAT3/STAT3 were no difference compared with model group, which indicated that SL blocked JAK2/STAT3 pathway owing to decreased the expression of total proteins of JAK2 and STAT3.

## Discussion

In the present study, we established a mouse MI/R model and conditioned medium transfer models of macrophages and cardiomyocytes. Results showed that SL treatment reduced infarct size and decreased myocardial tissue apoptosis. Meanwhile, SL reduced cardiomyocyte apoptosis in conditional medium transfer models of macrophages and cardiomyocytes. Furthermore, SL suppressed M1 macrophage polarization by down-regulating *miR-155* expression, promoting SOCS3 expression and blocking JAK2/STAT3 signalling pathway. Taken together, these observations suggest that SL could be a potential therapy in MI/RI.

Myocardial ischaemia-reperfusion injury refers to myocardial tissue injury induced by reperfusion therapy after myocardial infarction. Myocardial ischaemia reperfusion usually leads to failure of cardiac function, increased infarct size and disordered biochemical metabolism. The infarcted area is the most direct evaluation of cardiac function. CK level is viewed as a symbol of the effects and prognosis of MI/RI, lower activity of CK stands for an obvious protective effect after cardiac muscle injury (Apple 1989). LDH is also a biochemical index to diagnose ischaemic heart disease and was closely associated with heart disease development. During MI/R, timely blood perfusion results in reactive oxygen species (ROS) overproduction, SOD is the primary mediator of oxygen free radicals and can reduce the contents of ROS and alleviate oxidative stress in MI/R (Grill et al. 1992). In our experiment, the results proved that SL shrinks infarcted size, decreased CK, LDH levels and increases SOD activity, which illustrated SL attenuated MI/RI.

Myocardial cell apoptosis is viewed as an important mechanism of MI/RI. In the process of MI/RI, excessive ROS stimulates the opening of mitochondrial permeability transition pore, expedites the release of pro-apoptotic protein, Cytochrome c (Cyt c), into the cytosol, and then Cyt c can bind and activate pro-caspase-9. Eventually, caspase-9 and downstream protein, caspase-3, were activated and apoptosis occurs (Li et al. 2019). It is well known that the Bcl-2 protein family regulates mitochondrial-initiated apoptosis by taking part in the release of Cyt c, some of the Bcl-2 members are proapoptotic such as Bax, Bak, Bid, and Bim, while some have an anti-apoptotic function such as Bcl-2, Bcl-xL, and Bcl-W (Siddiqui et al. 2015). In our experiment, SL reduced the percent apoptosis of cardiomyocytes, downregulated the expression of pro-apoptotic proteins (cleaved caspase-3, cleaved caspase-9, Bax), and upregulated the expression of an anti-apoptotic protein (Bcl-2) in MI/R mice, meanwhile, in the conditional medium transfer models, our results also showed that SL reduced H/R cardiomyocytes apoptosis and rate of cleaved-caspase-3/caspase-3 and raised Bcl2/Bax ratio, which suggested SL reduced cardiomyocyte apoptosis in MI/R mice and H/R cardiomyocytes induced by M1 macrophages.

MI/R aggravates tissue injury by recruiting immune cells to the impaired tissues. Macrophages, as the predominant inflammatory cells in repairing myocardial tissue, can infiltrate the myocardium and settle for a long time (2 weeks) (Lafuse et al. 2020). Pro-inflammatory macrophages (M1 macrophages) mainly exist in the early stage of MI/RI and peak within 1–3 days. M1 macrophages increase the secretion of pro-inflammatory factors and chemokines, such as TNF-α, IL-1β, IL-6, CXCL1, CCL2 and CCR2, which leads to a strong pro-inflammatory reaction and contribute to MI/RI (Guicciardi et al. 2018). Thus, it is confirmed that inflammatory responses mediated by M1 macrophages are a critical reason for aggravating MI/R tissue damage. Importantly, inhibiting extravagant inflammatory by down-regulating M1 macrophages can reduce myocardial necrosis and decrease the infarct zone. Some monomer compounds of Traditional Chinese Medicine such as tanshinone II A and baicalin, have also been reported to reduce myocardial ischaemia-reperfusion injury by inhibiting M1 macrophage polarization (Hu et al. 2015; Xu et al. 2020). In the present study, our results also determined that SL obviously diminished iNOS/F4/80 and CD86/F4/80 positive cells ratio in MI/R mice and LPS-induced pMACs, which indicated that SL inhibited M1 macrophage polarization in MI/RI. Meanwhile, SL down-regulated the expression of pro-inflammatory factors (TNF-α, IL-1β, IL-6, CXCL1, CCL2, CCR2) and increased the expression of IL-10 *in vivo* and *in vitro*. Thus, we believed that SL inhibited the M1 macrophage polarization and reduced the inflammatory response in the process of MI/R.

*MiR-155* is a microRNA associated with inflammation and is highly expressed in activating macrophages. Particularly, *miR-155* has also been found to relate to MI/RI, endogenous suppression of *miR-155* reduces effectively myocardial infarct size, diminishes cardiomyocyte apoptosis, alleviates cardiac hypertrophy and remodelling, suppresses the development of heart failure and enhances the survival rate of rats, whereas *miR-155* knock-out protects the cardiac fibroblasts (He et al. 2016; Hu et al. 2019). Meanwhile, *miR-155*, as an LPS-responsive miRNA, is upregulated in macrophages and promotes the production of proinflammatory cytokines and increased *miRNA-155* expression results in the polarization of M1 macrophages (Jablonski et al. 2016; Pasca et al. 2020). SOCS3 is one of the target proteins of *miR-155* and *miR-155* suppresses SOCS3 expression (Wang et al. 2019). A previous study suggested that SOCS3 deficiency in macrophages promotes M1 macrophage polarization and inflammatory responses (Qin et al. 2012). In this study, we found that *miR-155* expression was up-regulated in MI/R mice as well as in LPS-stimulated pMACs, but the *miR-155* level was down-regulated after SL treatment. In addition, SL decreased *miR-155*/F4/80 and *miR-155*/CD86 positive makers in MI/R mice and M1 macrophages. Therefore, we speculated that SL blocked M1 macrophage-mediated pathological processes in MI/RI involving *miR-155* silence. Furthermore, SL increased gene and protein expression of SOCS3 level in LPS-treated macrophages, which is consistent with the inhibition effect of SL to *miR-155*. In addition, SOCS3 is a known prevailing inhibitor of JAK2/STAT3 signalling and can block JAK2 tyrosine kinase activity through the kinase inhibitory region (Inagaki-Ohara et al. 2013). The JAK2/STAT3 signalling pathway is viewed as a critical inflammatory mediator, in macrophages, JAK2 can dimerize and autophosphorylate to form p-JAK2, which further activates STAT3 into p-STAT3, then the activated p-STAT3 enhances transcription of inflammatory factors (Chen et al. 2019). To explore the second possible reason for the inhibitive effect of SL on inflammation, which is related to the JAK2/STAT3 pathway regulated by *miR-155*-targeting SOCS3, we examined protein expression of JAK2, p-JAK2, STAT3, p-STAT3. In this study, our results showed that the inhibitive effect of SL on of JAK2/STAT3 pathway was not decreased the ratio of p-JAK2/JAK2 and p-STAT3/STAT3, but diminished the expression of total proteins of JAK2 and STAT3. Therefore, we inferred that SL repressed inflammation in connection with JAK2/STAT3 signalling pathway blocking. However, the primary cause of the inhibitive effect of SL on the JAK2/STAT3 pathway could not be associated with up-regulated SOCS3 expression. Collectively, we believed that SL inhibited M1 macrophage polarization though reducing the expression of *miR-155* and increasing the level of SOCS3, thus reduce the inflammatory response during MI/R. Meanwhile, the inhibition of SL on inflammatory response may be related to blocking JAK2/STAT3 pathway and the underlying mechanism remains to be determined in future.

**Figure 10.**
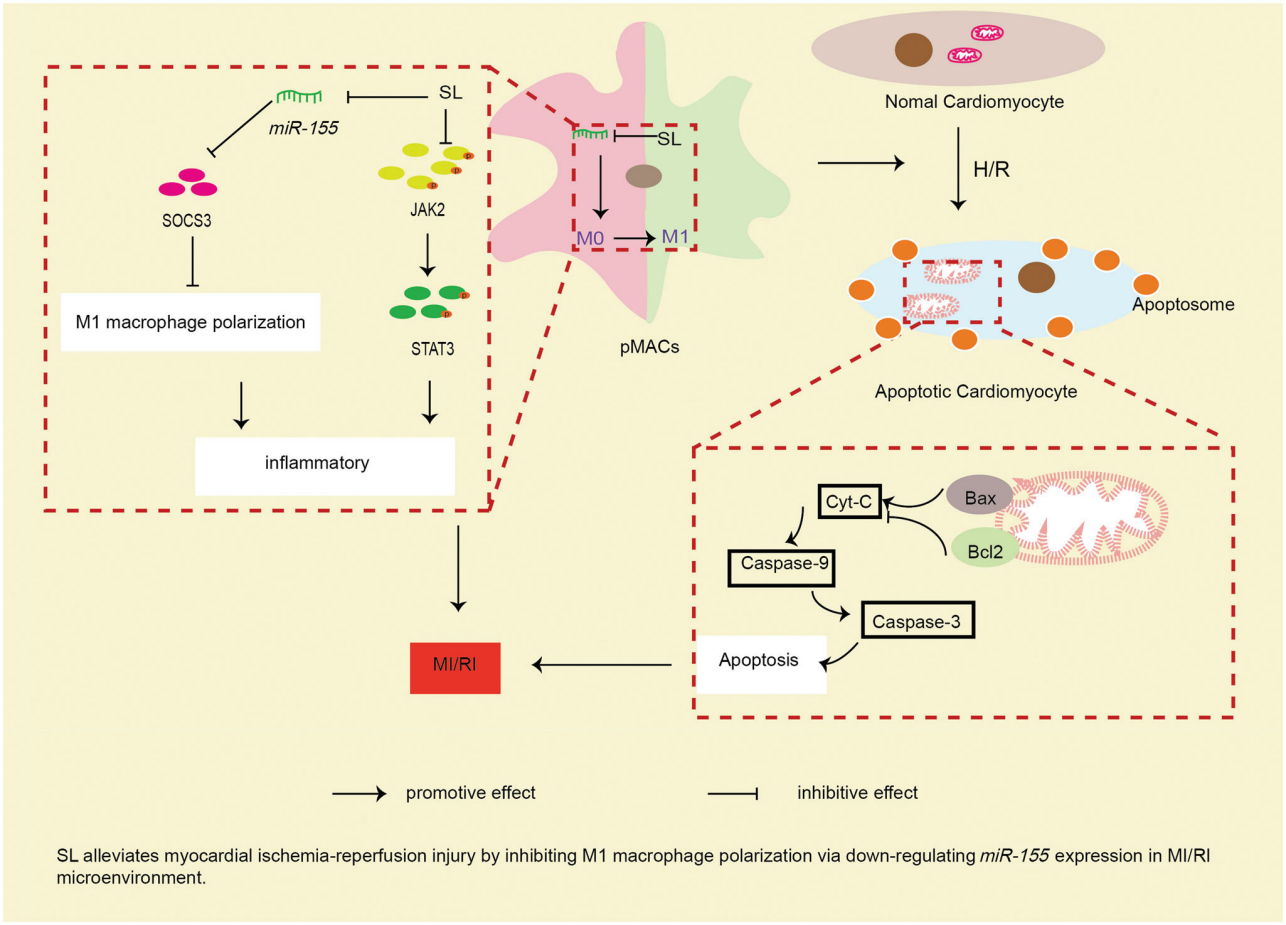
SL alleviates myocardial ischaemia-reperfusion injury by inhibiting M1 macrophage polarization via down-regulating *miR-155* expression in MI/RI microenvironment.

Through the results *in vivo* and *in vitro*, we believe that SL can reduce cardiomyocyte apoptosis and tissue damage in MI/RI by suppressing M1 macrophage polarization. In conclusion, the present study demonstrated that SL mitigates tissue damage and cell apoptosis in MI/RI by restricting M1 macrophage polarization and inflammation. The potential mechanism may be related to its effect on *miR-155* inhibition, SOCS3 up-regulation and JAK2/STAT3 signalling pathway blocking (Figure 10). These results also offer available evidence for potential therapy of SL in MI/RI.

## Authors’ contributions

Xiao Z, Ya W and Min S contributed to the conception of the study. Min S, Xi C, Jing Z, Jing Li, Yuan Z and Qing Y performed the experiment. Qi L, Yu L, Ying C, Xiao W and Wei C performed the data analyses. Min S, Ya W and Xiao Z wrote the manuscript.
